# Systematic review and integrated analysis of prognostic gene signatures for prostate cancer patients

**DOI:** 10.1007/s12672-023-00847-4

**Published:** 2023-12-19

**Authors:** Yang An, Wenyuan Lu, Shijia Li, Xiaoyan Lu, Yuanyuan Zhang, Dongcheng Han, Dingyuan Su, Jiaxin Jia, Jiaxin Yuan, Binbin Zhao, Mengjie Tu, Xinyu Li, Xiaoqing Wang, Na Fang, Shaoping Ji

**Affiliations:** 1 School of Basic Medical Sciences, Henan University, Kaifeng, 475004 China; 2 Department of Biochemistry and Molecular Biology, Cell Signal Transduction Laboratory, School of Basic Medical Sciences, Henan University, Jinming Street, Kaifeng, 475004 Henan China; 3 Henan Provincial Engineering Center for Tumor Molecular Medicine, Kaifeng Key Laboratory of Cell Signal Transduction, Kaifeng, 475004 China

**Keywords:** Gene signature, Prostate cancer, Prognosis, Biomarker, Survival

## Abstract

Prostate cancer (PC) is one of the most common cancers in men and becoming the second leading cause of cancer fatalities. At present, the lack of effective strategies for prognosis of PC patients is still a problem to be solved. Therefore, it is significant to identify potential gene signatures for PC patients’ prognosis. Here, we summarized 71 different prognostic gene signatures for PC and concluded 3 strategies for signature construction after extensive investigation. In addition, 14 genes frequently appeared in 71 different gene signatures, which enriched in mitotic and cell cycle. This review provides extensive understanding and integrated analysis of current prognostic signatures of PC, which may help researchers to construct gene signatures of PC and guide future clinical treatment.

## Introduction

**Fig. 1:**
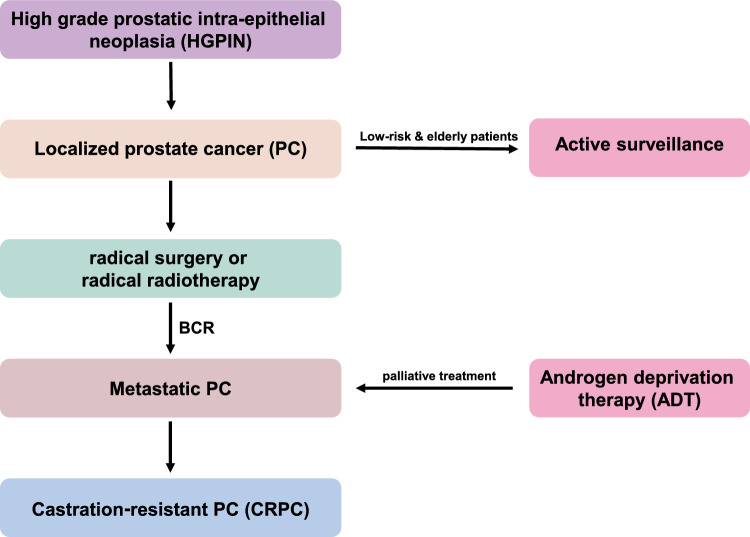
The development of prostate cancer

Prostate Cancer (PC) is one of the most common malignant tumors, and the incidence of PC has been on the increase in recent years, especially the late-stage PC [1]. Risk factors of PC mainly include age, genetic and family history, behavior and lifestyle, environmental factors [2]. PC arises from high grade intra-epithelial neoplasia and develops into localized PC [3–5]. In clinical practice, localized prostate cancer can be effectively treated, and even cured, through radical surgery or radiotherapy. Active surveillance may be chosen for some low-risk and elderly patients. However, some patients do not benefit from radical surgery or radiotherapy, but are afflicted with biochemical recurrence (BCR) [6, 7]. Once BCR occurs, patients of PC have a possibility to be liable to undergo metastasis [6, 8]. Androgen deprivation therapy (ADT) is a preferred treatment for patients with metastatic PC. Unfortunately, some diseases developed into castration-resistant PC after ADT treatment [6, 9, 10] (Fig. 1). Therefore, if risk factors of prognosis of PC patients could be accurately predicted, early intervention and targeted therapy could prevent the progression of PC, which will benefit more patients.

Recently, clinicians mainly take into account the clinical stages and clinicopathological features to guide the treatment of PC patients. However, due to the molecular heterogeneity of PC, some patients would be resistant to the uniform treatment and progress to recurrence [11–13]. Therefore, it is significant to identify prognostic biomarkers for PC patients to predict outcomes and guide treatment. To improve prognosis of PC patients, gene signatures are applied to predict survival outcome of PC patients by risk stratification, thus classifying patients as high or low risk. For instance, a 7-gene signature has been developed to distinguish indolent PC from aggressive PC [14]. In addition, a 20-gene signature has been constructed to identify patients at risk of metastatic progression after prostatectomy [15], and a 10-gene signature has been identified to predict the risk of BCR of PC patients [16]. Besides, gene signatures also have been applied to the personalized diagnosis and treatment of PC patients [17, 18].

Consequently, the aim of the present study was to conduct a comprehensive review and analysis of previously reported gene signatures that predict survival outcome of PC patients. In this review, 71 different gene signatures were summarized and 3 strategies for gene signature construction were concluded.

## Methods

### Data collection and selection

In order to evaluate previously reported gene signatures for PC, a total of 282 articles were included by searching PubMed database with keywords ‘‘prognostic gene signature AND prostate cancer’’. The dates for database search were September 20, 2020, and February 6, 2021, respectively. At first, a total of 279 articles were included (3 articles were not available). By selection, gene signatures based on mRNA expression profiling and correlated with patients' survival outcomes were included. For consistency, exclusion criteria was as follows: (i) Review or meta-analysis; (ii) not prostate cancer; (iii) gene signature not mentioned; (iv) signatures were not derived from mRNA expression profiling; (v) no prognostic analysis. The inclusion of relevant articles was executed according to the PICO (P: prostate cancer patients; I: prognostic gene signature; O: survival outcomes). As a result, 71 different gene signatures were summarized from 68 studies.

### Data interpretation and statistical analysis

To compare prognostic abilities of different gene signatures, data from training set was collected and summarized. Additionally, during the data extraction process, results from the TCGA database and data with overall survival (OS) were given priority. To evaluate these prognostic signature models, some indexes were summarized, including gene name, p value, AUC of ROC curve, Hazard ratio (HR) and 95% confidence interval (CI) estimated by Cox regression analysis (Table 1, 2, 3, 4). To visually demonstrate the ability of risk stratification of gene signature, we extracted HR and 95% CI from these articles and made a forest plot (Fig. 3) by R 4.3.1 (Among 71 gene signatures included in this study, only 31 gene signatures showed HR and 95% CI). In addition, robust genes were identified according to their usage frequency in 71 different gene signatures. Furthermore, pathway enrichment analysis of robust genes were conducted at www.metascape.org. Steps were as follows: (i) input a gene list; (ii) select species: H.sapiens; (iii) perform Express Analysis.

**Table 1:** Gene signature information of strategy I

Authors	Gene number	Gene name	Function of gene signature	Detection method	AUC of ROC	Survival event	Survival analysis		Multivariate analysis	
							HR 95% CI	*p* value	HR 95% CI	*P* value
Xiao Kefeng et al. [19]	8	PMP22, HPN, LMTK2, FN1, EZH2, GOLM1, PCA3, GSTP1	To distinguish aggressive PC	qRT-PCR	0.967					
Xiao Kefeng et al. [19]	5	CCND1, LMTK2, FN1, EZH2, GOLM1	Diagnosis	qRT-PCR	0.992					
Zhao ShuangG et al. [15]	20	NVL, CENPF, SMC4, CAMK2N1, STMN1, TPX2, MKI67, SMC2, NSMCE2, HDAC9, SQLE, LCLAT1, LPGAT1, TOP2A, MTFR1, MDH1B, NRP1, ERO1LB, PRKCA, POSTN	To identify patients at risk of metastatic progression after prostatectomy	Microarray qRT-PCR		MFS	7.3 (4.8–11.0)			
Rajan Prabhakar et al. [52]	7	ADAM7, FAM72B, BUB1B, CCNB1, CCNB2, TTK, CDK1	To identify high-risk prostate cancer	RNA-Seq		DFS		*P* < 0.015		
Bismar Tarek et al. [23]	10	ANXA4, Syntenin, WNT2, PLA2G7, CHD5, ANK3, FRZB, MEIS2, LEF1, ING3	To predict outcome of patients with PC	Bioinformatics		OS	2.38 (1.45–3.8)*	*p* < 0.001*	2.3 (1.1–4.6)	*p* = 0.016
Wu Chin-lee et al. [53]	32	ACTR3B, APOC1, ATP8A1, akaAPM2, akaCDC2, CDON, DEGS1, SYMN, DPP4, F12, FEV, GATA3, GSTM3, HIST1H3H, HOXC4, TMEM132A, IGSF1, IGSF6, INHBA, KRT15, LDHB, KIF18B, NCAPG2, MAOA, MT1F, OIP5, PPP3CB, QPRT, TPX2 (exclude three normalization genes)	To predict progression of PC	Bioinformatics/RT-PCR				*P* < 0.0001	4.05 (1.50–10.94)*	*p* = 0.0057*
Olmos David et al. [54]	9	HMBS, TMCC2, SNCA, SLC4A1, STOM, GABARAPL2, RIOK3, TERF2IP, TFDP1	To identify patients with mCRPC with poorer outcome	Microarray qRT-PCR		OS		*p* = 0.001	3.05 (1.22–7.64)	*p* = 0.017
Chen Xin et al. [14]	7	RRAGD, PQBP1, HIST1H2BC, ALDH1A2, TRIM22, RBPMS, HSPB8	To distinguish indolent PC from those that will relapse	Microarray		DFS	2.6*	*p* = 0.035*		
Planche Anne et al. [55]	36	PRAC, ASPN, CTHRC1, TGRP, AGR2, POSTN, ESR1, NKX31, HOXB13, SFRP1, BMPR1B, FOLH1, RSPO3, PKP2, ERG, TSPAN1, HOXC6, GREB1, NELL2, BMP5, PENK, GPM6A, DKK1, PTGS1, SEMA3E, FOXQ1, DPT, ARHGAP28, HOXD13, TSLP, PRKCB1, PTGDS, HAPLN1, GPR133, PGF, HOXD11	To predict clinical outcome	Microarray qRT-PCR		OS		*p* = 0.00234**		
Sinnott Jennifer et al. [56]	30	ACTG2, SLC22A3, MYH11, ANO7, TPM2, CSRP1, MIR21, PCP4, SYNM, MYL9, CNN1, MYBPC1, SYNPO2, MYLK, BTG2, DES, ACTA2, SERPINA3, SOD2, DPP4, FLNA, NR4A1, ITGA5, SORBS1, MSMB, GLB1L3, NCAPD3, MT2A, ASPN, ZFP36	To improve lethal result prediction among men with GS = 7	Microarray	0.73**					
Mo Fan et al. [57]	93	See Supplemental Table	To distinguish indolent PC from aggressive PC	Microarray	0.86	MFS		*p* < 0.001*		
Mangiola Stefano et al. [58]	3	IGHAI, OLFM4, RERGL	To diagnose high-risk PC	qQT-PCR	0.72*					
Xu Ning et al. [25]	4	HOXB5, GPC2, PGA5, AMBN	To predict survival of PC	Bioinformatics	0.904	OS		*p* = 0.00822		
SchmidtLinnea et al. [59]	19	PAGE4, KCTD14, KIAA1210, KRT23, TGM4, RP3-523K23.2, KRT5, C20orf166-AS1, MIR205HG, RP11-746E8.1, SMOC1, COL13A1, RBFOX3, RP11-160A10.2, FAM83B, FLRT3, MUC4, KRT15, HEATR8	For PC risk stratification	RNA-Seq		RFS		*p* < 0.001**	2.09(1.29–3.37)**	*p* = 0.0026**
SchmidtLinnea et al. [59]	20	SYT4, ANKRD30A, PARP15, RP11.242D8.1, CTD-2536I1.1, RP11-68I3.11, GTF2H3, VARS, IL7R, DAPP1, NTNG1, SP140L, RP11-137L10.6, LY75, ZNF599, RP11-366M4.3, ENPP2, ASB5, RP11-44F14.8, EXTL3-AS1	For PC risk stratification	RNA-Seq		RFS		*p* < 0.001**	2.01(1.51–2.69)**	*p* < 0.0001**
Mu Haiqi et al. [50]	9	GART, RRM2, CACNA1D, PIK3R1, MYH11, MYLK, TNC, MYL9, KCNMA1	To predict prognosis of PC	Bioinformatics	0.76*	OS	5.33 (1.12–25.39)*	*p* = 0.03561*		
Liu Shuang et al. [60]	7	BCO1, BAIAP2L2, C7, AP000844.2, ASB9, MKI67P1, TMEM272	For early prognosis and diagnosis	Bioinformatics	1 year = 0.995, 3 year = 0.886, 5 year = 0.812, 10 year = 0.606	OS		*p* = 0.007706		
Shao Ning et al. [17]	6	ZNF467, SH3 RF2, PPFIA2, MYT1, TROAP, GOLGA7B	To predict prognosis of PC	Bioinformatics/microarray	1 year = 0.74, 2 year = 0.73 5 year = 0.74	RFS	0.28 (0.18–0.44)	*P* < 0.001		
Wu Xiangkun et al. [16]	10	SEMG2, KCNJ16, TFAP2B, SYCE1. KCNU1, AFP, GUCY1B2, GRIA4, NXPH1, SOX11	To predict BCR risk	Bioinformatics	3 year = 0.791, 5 year = 0.835	RFS	5.18 (3.214–8.272)	*p* < 0.0001	3.34 (1.87–6.00)	*p* < 0.0001
Chipidza Fallon et al. [6]	186	See Supplemental Table	For PC risk stratification	Bioinformatics	0.84	MFS	2.55 (1.7–3.9)	*p* < 0.0001	1.60 (1.05–2.44)*	*p* = 0.02*
Yuan Penghui et al. [24]	4	ANO4, EZH2, PARM1, SRD5A2	To predict prognosis of PC	Bioinformatics	1 year = 0.638* 3 year = 0.723* 5 year = 0.662*	RFS		*p* = 0.00013*		
Hou Qi et al. [61]	15	FKRP, SEL1L, IGFALS, PNMA2, ARHGAP8, PHF3, HMG20B, LPHN3, NPY, EPHB2, NNMY, C1QTNF3, FABP5, MTRR, ITPR1	For diagnostic and prognostic	Bioinformatics	0.808*	OS	2.462 (1.224–4.952)*	*p* = 0.003*		
Pang Xiaocong et al. [27]	6	SPP1, CEACAM6, COL15A1, FN1, POSTN, ARG1	To distinguish mCRPC from HSPC	Bioinformatics/RT-qPCR	0.849					
Zhao yue et al. [62]	5	AR, PDHA1, RAN, DPP4, DAZAP1	To predict the prognosis of PC	Bioinformatics	0.745	OS		*p* = 0.0014	1.00 (1.00–1.00)	*p* = 0.002
Xu Jiaju et al. [51]	5	HNRNPA2B1, NXF1, RBMX, YTHDF1, TRMT112	To predict malignant prognosis	Bioinformatics	0.716	DFS		*p* < 0.001	1.074 (1.003–1.151)	*p* = 0.042
Marin-Aguilera Mercedes et al. [63]	2	SELENBP1, MMP9	To identify more aggressive clinical behavior	RT-PCR	0.79	OS	9.72 (3.62–26.06)	*P* < 0.001	14.53 (1.91–110.75)	*p* = 0.0098
Li Chirong*et al*. [64]	3	PDCD4, KLF6, ABCG1	To predict BCR	RT-PCR		RFS	9.7 (2.5–37.3)	*p* = 0.0009	20.234 (2.613–156.684)*	*p* = 0.002*
Georgescu Constantin et al. [65]	11	BUB1B, CDC45, CDK1, CENPI, CLSPN, ERCC6L, EXO1, NCAPG, NUSAR1, RAD51, RRM2	Risk stratification	qRT-PCR					2.27 (1.05–4.91)*	*p* = 0.038*
Ong Chee et al. [26]	35	ACTA2, ACTG2, MYH11, TPM2, ROCK2, COX4I1, UBA52, TPT1, TRPM4, DOPEY2, RNY1, CLTB, TOMM7, KLK3, RPS10, LRRC26, ATP9A, CDC37, SUMF2, SNORA61, CLUAP1, PHGDH, SL44A4, YIF1A, AKT1, RPL9, RPS29, RNU1G3, LILRB3, HGS, SERPINB6, CCR6, RPS19, FASN, VIPR1	Risk stratification in intermediate risk PC cases	Microarraybioinformatics	0.719*				6.95 (2.73–17.54)*	*p* < 0.0001*
Gu Yan et al. [6]	27	HAGHL, LCN12, DCST2, VGF, RGS11, PRR7, LINC01089, MXD3, BIRC5, LTC4S, H1FX-AS1, FPR3, RAB30, RIPOR2, NOD2, PLXNA4, RRAGC, TFX10C, PI15, ZFHX4, LAMP3, HDAC9, MCTP1, KCNN3, PCDHB8, PCDHGB2, PCDHGA5	To predict PC relapse	RT-PCR/bioinformatics	0.88**	DFS	2.72 (2.22–3.33)	*p* < 0.001	2.32 (1.88–2.88)	*p* = 5.74 × 10^–15^
Li Ping et al. [66]	4	PHYHD1, CENPF, ALDH2, GDF15	To predict PFS and MRI visibility of PC	RT-PCR/bioinformatics	0.86*	BFS	2.09 (1.27–3.44)*	*P* = 0.003*	2.53 (1.55–4.11)*	*p* < 0.001
Jhun Min et al. [67]	49	PDGFB, ASPN, FOXS1, SMC4, FAM72B, ITGBL1, LPPR4, SPAG1, BUB1, GOLGA7B, CENPF, GDF3, MAPK8IP2, ESM1, PRC1, MYT1, LRFN2, SHCBP1, AHRR, CBX2, GMNN, NUF2, STC2, RAI14, FGF14, ZNF467, TMEM132E, FAM72D, CST2, KIF14, APLNR, DLGAP5, CENPE, IGSF1, NAAA, ASPA, TAOK3, SLC22A1, C2orf88, NCAPD3, GLB1L3, PAGE4, ANO7, EDN3, TPT1, ADPGK, PACSIN3, GLB1L2, PLOD1	To predict ML progression and recurrence of PC	RT-PCR/bioinformatics	0.68*	RFS	1.51 (1.24–1.82)*	*P* = 2.7 × 10^–5^*		

PC, prostate cancer; mCRPC, metastatic castration-resistant prostate cancer; HSPC, hormone sensitive prostate cancer; PFS, progression-free survival, MFS, metastasis-free survival; RFS, recurrence-free survival; BFS, Biochemical recurrence(BCR)‐free survival; DFS, Disease-free survival; ML, metastatic-lethal; GS, gleason score; data from validation set are marked with*; data from test set are marked with**

**Table 2:** Gene signature information of strategy II

Authors	Gene number	Gene name	Function of gene signature	Detection method	AUC Of ROC	Survival event	Survival analysis		Multivariate COX analysis	
							HR (95% CI)	*p* value	HR (95% CI)	*P* value
Wu Lei et al. [68]	6	FADD, GABARAPL2, MYC, RAB24, RUBCN, NPC1	To predict OS of PC	Bioinformatics	0.928	OS		*p* = 0.0148		
Cheng Yifei et al. [69]	3	BNIP3, NPC1, TP53	To predict prognosis of PC	Bioinformatics	0.9364	OS		*p* = 1.31 × 10^–3^	3.25 (1.678–6.296)	*p* < 0.001
Cheng Yifei et al. [69]	5	ATG9B, DNAJB1, HSPB8, NKX2-3, TP63	To predict risk of PC	Bioinformatics	0.9858					
Hu Daixing et al. [33]	5	FAM215A, FADD, MYC, RHEB, ATG16L1	To predict and prognosis OS of PC	Bioinformatics	0.84	OS	6.391 (1.581–25.840)	*p* = 5.005 × 10^–3^		
Hu Daixing et al. [33]	22	ULK2, NLRC4, MAPK1, ATG4D, MAPK3, ATG2A, ATG9B, FOXO1, PTEN, HDAC6, PRKN, HSPB8, P4HB, MAP2K7, MTOR, RHEB, TSC1, BIRC5, RGS19, RAB24, PTK6, NRG2	To predict and prognosis DFS of PC	Bioinformatics	0.85	DFS	7.407 (4.850–11.320)	*p* = 4.441 × 10^–16^		
Ekanem Titus et al. [35]	3	CDK4, TWIST1, SNAI2	Marker of glycidamide exposure and to predict outcome of PC	Bioinformatics		OS	7.83 (3.71–16.54)	*p* = 6.876 × 10^–8^		
Zhang Yanlong et al. [18]	6	CACNG4, SLC2A4, EPHX2, CA14, NUDT7, ADH5	To guide prognosis, diagnosis and treatment of PC	Bioinformatics	1 year = 0.710; 3 year = 0.705; 5 year = 0.698	DFS		*p* = 1.997 × 10^–7^	1.77 (1.34–2.34)	*p* = 0.00007
Yang Lingjian et al. [70]	28	ADAMTS4, ATF3, BHLHE40, BTG2, CSRNP, CYR61, EGR1, EGR2, EGR3, FOSB, FOSL2, GEM, JUNB, KLF10, KLF6, LIF, MCL1, NR4A3, PPP1R15A, RHOB, SELE, SIK1, SLC2A14, SLC2A3, SOCS3, THBS1, TIPARP, ZFP36	To assess the prognosis of PC patients	Bioinformatics	0.79*	BFS		*p* = 1.9 × 10^–8^	3.51 (1.21–10.15)*	*p* = 0.021*
Wang Jiamin et al. [71]	2	METTL14, YTHDF2	Prognostic stratification of PC	Bioinformatics	3 year = 0.75; 5 year = 0.762	OS		*p* = 1.252 × 10^–3^	6.318 (1.230–32.467)	*p* = 0.027
Cao Zhexu et al. [72]	6	LUC7L3, SUGP2, SF3B1, CST3, UBAP2, ARHGEF39	to stratify risk of PC patients	Bioinformatic/ qRT-PCR	0.794	OS		*p* = 0.00104		
Roberto Domenica et al. [73]	5	HSD17B4, SRD5A2, RPTOR, PGRMC1, CCNE2	To distinguish G3 from G4	RT‐PCR	0.83					
Irshad Shazia et al. [74]	3	FGFR1, PMP22, CDKN1A	To predict outcome of indolent PC	Bioinformatics		BFS		*p* = 0.005*		*p* = 0.042#*
Zhang Qijie et al. [30]	15	RAB27A, HSF1, BTG2, AURKB, TPT1, NLRP12, PHLDA3, CDKN2A, STX4, E2F1, NSMF, MSX1, ADGRB1, MT1F, ERP29	To predict BCR	Bioinformatics /qRT-PCR	0.899 (year1-5,mean)	RFS			7.826 (3.516–17.415)	
Ragnum H et al. [75]	32	See Supplemental Table	To predict aggressive PC	Bioinformatics/microarray		OS		*p* < 0.001	3.17 (1.45–6.93)*	*p* = 0.004 *
FreedlandStephen et al. [76]	31	See Supplemental Table, CCP gene signature	To identify high-risk patients undergoing EBRT	RT-PCR		RFS	2.55 (1.43–4.55)	*p* = 0.0017	2.11 (1.05–4.25)*	*p* = 0.034*
Cuzick Jack et al. [77]	31	See Supplemental Table, CCP gene signature	To predict death from PC	RT-PCR					1.65 (1.31–2.09)	*P* = 3 × 10^–5^
Cuzick Jack et al. [29]	31	See Supplemental Table, CCP gene signature	To predict outcome of PC	qRT-PCR	0.842 (clinical score + CCP score)	RFS			1.77 (1.40–2.22)	*p* = 4.3 × 10^–6^
Liu Bide et al. [11]	16	STK36, BOD1, CTNNBIP1, NAMPT, RBPJL, WNT4, BMP8B, GREM1, TCF15, LRP2, IFNG, IL17B, SEL1L, LATS2, PRKACB, FZD5	To predict early BCR	Bioinformatics	1 year = 0.819; 3 year = 0.791; 5 year = 0.818	RFS	4.91 (2.75–8.76)	*p* < 0.001		
Zhang Yao et al. [78]	22	NUP85, NUP35, NUP107, ASNS, MYEF2, FANCI, MAD2L1, OIP5, CDC7, AURKA, PIGA, TFRC, E2F1, SMC4, CDK1, PGM5, IGF1, NUP62, RAE1, DUSP12, SLC44A1, INS	To predict prognosis and disease progression of PC	Bioinformatics/RT-qPCR	0.776	BFS/RFS		BFS: *p* = 0.002 RFS: *p* < 0.001	1.05 (1.00–1.10)	*p* = 0.044
Zhang Yao et al. [78]	26	NUP85, NUP35, NUP107, ASNS, MYEF2, FANCI, MAD2L1, OIP5, CDC7, AURKA, PIGA, TFRC, E2F1, SMC4, CDK1, PGM5, IGF1, ATF5, GCG, RORC, GPX7, RGN, C1QTNF1, POU2F2, COMT, GSK3A	To predict prognosis and disease progression of PC	Bioinformatics/RT-qPCR	0.780	BFS/RFS		BFS: *p* < 0.001 RFS: *P* < 0.001	1.06 (1.01–1.11)	*p* = 0.030
Gao Lei et al. [79]	6	MSI1, MBNL2, LENG9, REXO2, RNASE1, PABPC1L	To predict PC prognosis	Bioinformatics	1 year = 0.799; 3 year = 0.736; 5 year = 0.714	RFS		*p* < 0.0001	1.19 (1.12–1.26)	*p* < 0.001
Jin Yaojian et al. [80]	14	AP3B1, DHX58, EGR1, IRF7, MAFB, FANCC, NKIRAS1, GPSM1, RORC, IRF9, PURA, MARCH5, MTA1, TP53	To predict PC recurrence	Bioinformatics	1 year = 0.87; 3 year = 0.95; 5 year = 0.89	RFS		*p* < 0.0001	10.55 (5.315–20.94)	*p* < 0.0001
Valcarcel-Jimenez Lorea et al. [81]	2	CRYAB, MITF	To assess the prognosis of PC patients	Bioinformatics/qRT-PCR		DFS	1.7 (1.1–2.9)	*p* = 0.0086		
Nim Hieu et al. [82]	2	CYP26A1, RDH10	To assess the possibility of PC relapse	Bioinformatics		DFS	2.213 (1.1–4.098)	*P* = 0.0248		
Zhao Shuang G et al. [83]	24	Unavailable	To assess response to postoperative radiotherapy of PC patients	Microarray		Cumulative incidence of distant metastasis	High PORTOS: 0.12 (0.03–0.41) Low PORTOS: 2.5 (1.6–4.1)	High PORTOS: *p* < 0.0001 Low PORTOS: *p* < 0.0001		
Zhang Shengping et al. [84]	43	See Supplemental Table	To predict the prognosis of PC patients	Bioinformatics	0.696					
Lin Dong et al. [85]	32	See Supplemental Table	to discriminate indolent PC from aggressive	Microarray		DFS		*P* = 0.01*		
Lin Dong et al. [85]	4	PRODH, OAT, ALDH4A1, GLUL	to discriminate indolent PC from aggressive	Microarray		DFS		*P* = 0.0001*		

PC, prostate cancer; OS, overall survival; RFS, Recurrence‐free survival; DFS, Disease-free survival; BFS, Biochemical recurrence(BCR)‐free survival; DMFS, Distant metastasis (DMET)-free survival; mCRPC, metastatic castration-resistant prostate cancer; *means data is derived from validation cohort. 0.042# means FGFR1/PMP22/CDKN1A three-gene signature together with GS in multivariate cox analysis; EBRT, External Beam Radiation Therapy

**Table 3:** Gene signature information of strategy III

Authors	Gene number	Gene name	Function of gene signature	Detection method	AUC Of ROC	Survival event	Survival analysis		Multivariate COX analysis	
							HR (95% CI)	*p* value	HR (95% CI)	*P* value
Kwan Edmond et al. [86]	4	AR-V7, GRHL2, HOXB13, FOXA1	To stratify risk in mCRPC	RT-PCR		OS		*p* = 0.0021	2.1 (1.1–4.0)	*p* = 0.019
Stelloo Suzan et al. [45]	9	DNER, EXT2, AMOTL1, RBM33, ZBTB20, XBP1, PMFBP1, HSD17B14, KLF9	To identify patients at risk of metastatic relapse after local–regional treatment	ChIP–qPCR	0.86	BFS/MFS		BFS: *p* = 0.00084 MFS: *p* = 0.049		
Chen Xin et al. [41]	2	FECH, CROT	To predict recurrence of PC	Bioinformatics		RFS	0.48	*p* = 0.037		
Magani Fiorella et al. [38]	7	KIF20A, KIF23, TOP2A, CCNB1, CCNB2, BUB1, BUB1B	To predict DFS of PC patients	RT-qPCR		DFS/OS		DFS:* p* = 0.0009 OS: *p* = 0.0026		
UrbanucciAlfonso et al. [39]	10	LAD1, COLEC12, KAT2A, FEN1, HSPH1, ZDHHC11, EEF1A2, ST3GAL1, HNRNPAB, CLDN3	To stratify risk of PC patients	IHC/qRT-PCR		BFS		*p* = 0.008*		

*PC* prostate cancer, *AR-V7* AR splice variant 7; OS, overall survival; RFS, Recurrence‐free survival; DFS, Disease-free survival; BFS, Biochemical recurrence(BCR)‐free survival; MFS, Metastasis-free survival; *means data is derived from validation cohort

**Table 4:** Gene signature information of other strategies

Authors	Gene number	Gene name	Function of gene signature	Detection method	AUC Of ROC	Survival event	Survival analysis		Multivariate COX analysis	
							HR (95% CI)	*p* value	HR (95% CI)	*P* value
Vittrant Benjamin et al. [87]	3	JUN, HES4, PPDPF	To predict BCR	Bioinformatics	0.761					
Cho Hyungseok et al. [48]	6	AR, AR-V7, PSA, PSMA, EpCAM, KRTI9	To predict metastatic PC	ddPCR	0.9					
Li Xiang et al. [46]	74 gene pairs (60 genes)	See Supplemental Table	For BFS prediction of PC patients after receiving RP	Bioinformatics/microarray		BFS	63.23 (18.56–215.40)	*p* < 2.2 × 10^–16^	41.17	*p* = 3.81 × 10^–8^
Shi Run et al. [47]	9	ALDH1A2, ASNS, SSTR1, FAM171B, FREM2, RSPO2, SRD5A2, TRIM14, VPS4A	To predict BFS of PC patients after receiving RP	Bioinformatics/microarray	0.836	BFS	5.787	*p* < 0.0001	5.084	*p* < 0.0001
Karnes R et al. [88]	49	See Supplemental Table	To assess the prognosis of PC patients receiving ADT after RP	Bioinformatics	0.84	Cumulative incidence of metastasis		Low ADT-RS: *p* = 0.989 High ADT-RS: *p* = 0.021	0.18 (0.05–0.65)*	*p* = 0.009*
Peng Z et al. [89]	3	VGLL3, IGFBP3, F3	For estimating overall survival	qPCR/RT-PCR	0.815(clinical parameters + the ESCGP signature)	OS	5.86 (2.91–11.78)	*p* < 0.001		
Glinsky Gennadi et al. [90]	11	Gbx2, KI67, CCNB1, BUB1, KNTC2, USP22, HCFC1, RNF2, ANK3, FGFR2, CES1	To predict therapy failure	Microarray		DFS	3.74 (3.010–25.83)	*p* < 0.0001		
Mazzu Ying et al. [91]	12	ANLN, BIRC5, CCNB1, CDC20, CDC6, CENPF, CEP55, KIF2C, MELK, MKI67, PTTG1, UBE2T	To identify aggressive subtypes of PC	Bioinformatics		DFS	3.42 (1.83–6.41)	*p* < 0.0001		
Walker Steven et al. [92]	70	See Supplemental Table	To detect metastatic recurrence risk in PC patients after radical prostatectomy	Bioinformatics/microarray	99.1 (98.5–99.8)	BCR/metastatic disease	BCR:1.76 (1.18–2.64)* metastatic disease: 3.47 (1.70–7.07)*	BCR: *p* = 0.0008* metastatic disease: *p* < 0.0001*	BCR:1.62 (1.13–2.33)* metastatic disease: 3.20 (1.76–5.80)*	BCR: *p* = 0.0092* metastatic disease: *p* = 0.0001*

*PC* prostate cancer, *RP* radical prostatectomy, *ADT* androgen deprivation therapy, *BFS* biochemical recurrence-free survival, *DFS* Disease-free survival, *BC*R biochemical recurrence

*Means data is derived from validation cohort

## Results

**Fig. 2:**
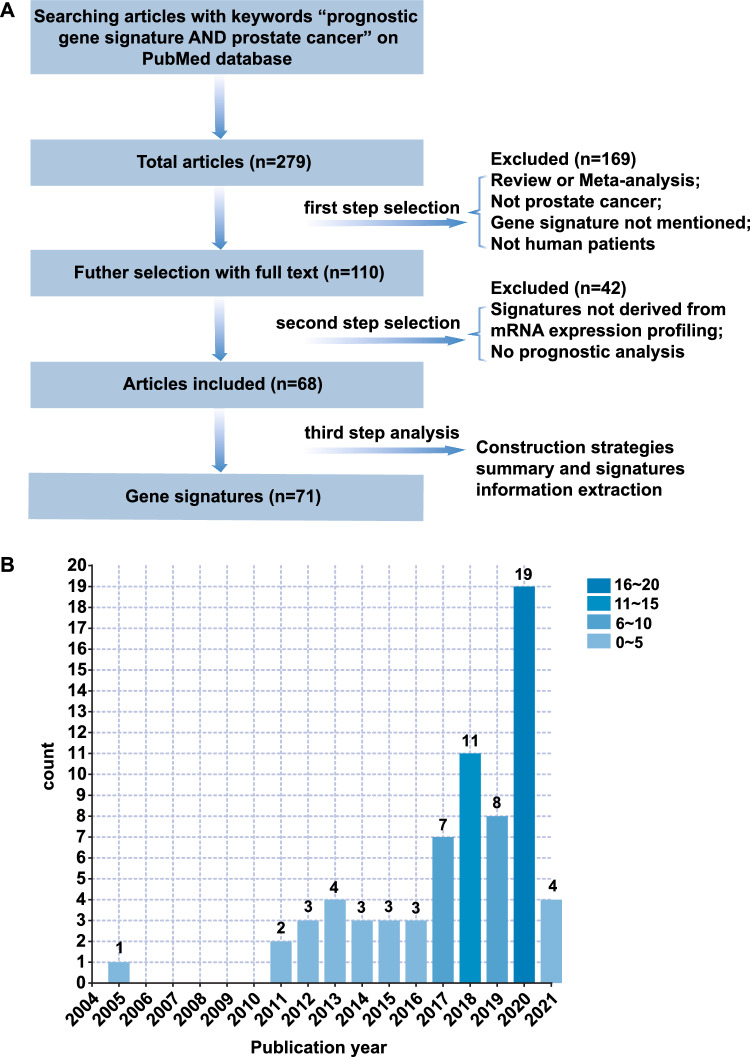
Data collection and interpretation. **A** Workflow of articles screening. **B** Publication year of 68 studies from 2005 to 2021

**Fig. 3:**
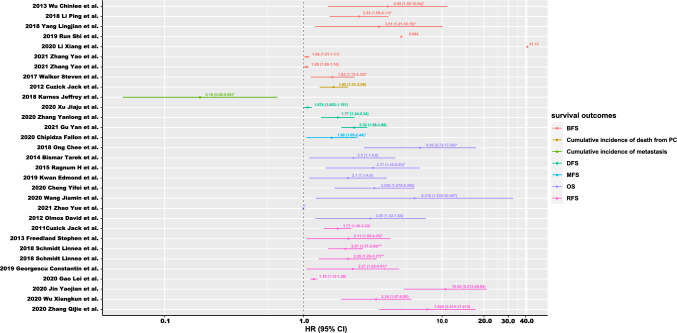
Estimate of 31 gene signatures via Meta-analysis. Forest plot, HR from validation set marked with*; HR from test set marked with**

By screening, 71 different gene signatures were summarized from 68 studies, which were published from 2005 to 2021 (Fig. 2). Comprehensive gene signature information related to survival of PC patients was presented in Table 1, 2, 3, 4 and Fig. 3. These signatures were associated with metastasis-free survival (MFS), overall survival (OS), disease-free survival (DFS), biochemical recurrence‐free survival (BFS) or recurrence-free survival (RFS) of PC patients (Tables 1, 2, 3, 4). In summary, methods of gene signature construction were mainly divided into three strategies according to different sources of prognostic genes. In Strategy I, gene signatures were constructed based on differentially expressed genes (DEGs); Strategy II was based on cellular process-related genes; Strategy III was based on AR (androgen receptor) or AR-Vs (androgen receptor variants)-related genes. In addition to the classification of gene signature construction, we also identified 14 robust genes from 71 different gene signatures.

### Strategy I: gene signatures based on DEGs

In strategy I, authors developed gene signatures on the basis of DEGs derived from different analysis methods. Shao N et al. (2020) obtained DEGs by comparing microarray data of PC samples with Gleason score (GS > 8 or GS < 6), and then identified 6 genes significantly related to biochemical recurrence (BCR) by using Lasso and Cox regression models [17]. In order to distinguish high-risk invasive PC from low-risk indolent PC, Xiao K et al. (2016) screened 8 DEGs between invasive and indolent PC using expression profiling of 87 prostatectomy samples [19]. Significantly, ETS (E26) fusion has been identified as a molecular subtype specific for PC [20–22]. Therefore, Bismar TA et al. (2014) screened 10 genes with significant differences between ERG fusion negative and positive samples as a 10-gene signature through singular value decomposition (SVD) analysis [23].

**Fig. 4:**
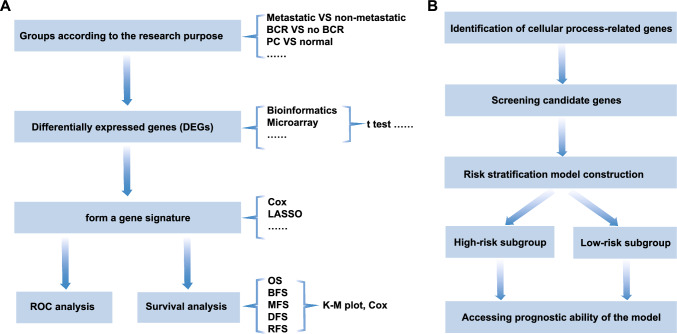
The flow chart of strategy I and II. **A** Strategy I: gene signatures based on DEGs. **B** Strategy II: gene signatures based on cellular process-related gene

The workflow of strategy I was summarized as follows: [1] Patient groups classified according to the research purpose. To study genes associated with PC metastasis, for instance, researchers divided PC samples into metastatic and non-metastatic groups [24]. (2) Acquisition of DEGs. Microarray or bioinformatics technique was used to analyze the gene expression profiling of different groups, and DEGs were obtained according to the criteria set at the beginning of these studies. (3) Establishment of a gene signature. Multivariate Cox regression model was used to screen genes significantly related to survival of PC patients from DEGs, thus a gene signature was constructed. (4) Validation. Survival and ROC analysis were performed on the established gene signature in other cohorts (Fig. 4A).

For instance, to predict survival outcome of PC patients, Xu N et al. (2018) analyzed 1417 DEGs by comparing expression profiling of PC and non-PC tissues, and then screened out 4 DEGs (HOXB5, GPC2, PGA5 and AMBN) through univariate and multivariate Cox regression analysis, which were significantly correlated with OS of PC patients [25]. Finally, multiple Cox regression coefficients corresponding to the four genes were multiplied by their corresponding gene expression levels and then summed to develop risk score. To confirm the predictive ability of a gene signature in PC, patients were divided into high-risk and low-risk groups and then subjected to Kaplan–Meier survival analysis. Furthermore, ROC curve analysis was applied to detect the discriminability of this gene signature [25]. More gene signature-related information of strategy I were presented in Table 1.

As for the strategy of developing gene signature through DEGs, some novel methods to identify DEGs have been applied. As Ong CW et al. (2018) described, through immunohistochemistry (IHC) to stratify intermediate risk PC, 35 DEGs related to high PTEN expression were identified to establish a signature [26]. As Pang X et al. (2019) described, DEGs were obtained through comparing expression profiling of hormone sensitive PC (HSPC) *vs*. metastatic castration-resistant PC (mCRPC), then extracellular matrix genes were enriched by performing biological pathway analysis. Thus, a gene signature composed of six genes was identified by correlation analysis of extracellular matrix genes [27]. In brief, as the fundamental feature of strategy I, gene signatures established through DEGs combined with other factors (such as PTEN expression or therapy sensitivity) also included.

### Strategy II: gene signatures based on cellular process-related genes

In this strategy, gene signatures were constructed based on cellular process-related genes, which were associated with progression of PC, including metabolism, CCP (cell cycle progression), apoptosis and autophagy. Zhang Y et al. (2020) considered that the unrestricted amplification feature of cancer cells would make the metabolic state of tumor tissues different from that of normal tissues. Thus, they developed a metabolism-associated 6-gene signature to guide diagnosis and DFS prediction of PC patients [18]. The expression of CCP-related genes fluctuated with cell cycle progression which may represent an aspect of tumor biology [28]. Thus, Cuzick J et al. (2011) employed 31 CCP genes to form a gene signature for predicting RFS of PC patients [29]. By literature retrospect, Zhang Q et al. (2020) declared that apoptosis is involved in the recurrence and progression of PC, thus they constructed an apoptosis-related gene signature for BCR prediction [30].

The major steps of Strategy II workflow were as follows: (1) To confirm that cellular process (such as metabolism, CCP, apoptosis or autophagy) is associated with PC progression. (2) Screening for genes associated with survival outcomes from cellular process-related genes. (3) Prognostic risk stratification model construction. (4) Verification of the prognostic risk model (Fig. 4B).

As known, autophagy leads to the degradation and recycling of intracellular components to maintain cellular homeostasis [31]. However, excessive autophagy may contribute to the elevated tumor invasion and lead to PC progression [32]. Thus, Hu D et al. (2020) constructed OS- and DFS-associated prognostic models based on autophagy-related genes (ARGs) [33]. First, differentially expressed genes were identified from 234 ARGs. Then, hub ARGs were screened using Cox regression analysis to construct a prognostic model. Finally, the correlation between clinicopathological features and this prognostic model was analyzed [33]. Glycidamide (GA) is known to be associated with malignant transformation of tumors [34, 35], however, little is known about which genes are induced by GA. Titus et al. (2019) demonstrated that GA accelerates migratory and growth ability of PC cells through influencing regulators of cell cycle and epithelial-to-mesenchymal transition (EMT). Hence, they constructed a 3-gene signature (CDK4, TWISTI and SNAI2) to predict survival outcome of PC patients upon GA exposure [35].

Gene signature derived from cellular process-related genes was the typical characteristics of this strategy. Therefore, we suspect that if the cellular process (such as metabolism, CCP, apoptosis or autophagy) has a greater impact on PC, the predictive ability of these gene signatures may be stronger than that of gene signature formed by other strategies. More gene signature related information of Strategy II was presented in Table 2.

### Strategy III: gene signatures based on AR or AR-Vs

**Fig. 5:**
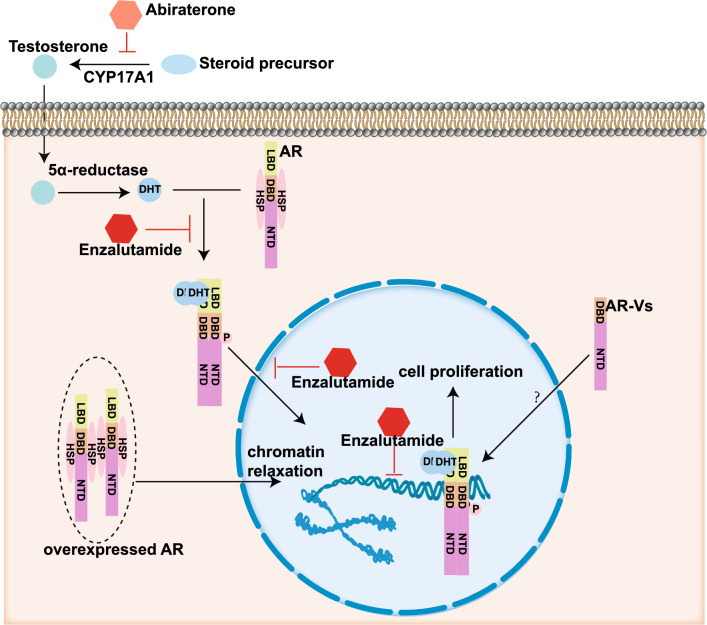
The effect of AR and AR-Vs on prostate cancer cells. Catalyzed by CYP17A1, steroids precursor turns into testosterone. After entering cells, testosterone transforms into dihydrotestosterone (DHT) by 5α-reductase. AR separates with its chaperones and binds to DHT, subsequently, phosphorylation occurs. After binding, AR and DHT enter nucleus in the form of dimer and bind to the corresponding site to promote cell proliferation. These processes can be blocked by Abiraterone and Enzalutamide, respectively. AR-Vs lack ligand-binding domain (LBD), but retain N-terminal domain (NTD) and DNA-binding domain (DBD). Some researchers have demonstrated that high expression of AR leads to chromatin relaxation, which is related to ADT resistance. It has also been suggested that ADT resistance may be related to the continuous activation of proto-oncogenes by AR-Vs, but the specific mechanism remains unclear [36–39]. This figure was graphed by Illustrator

**Fig. 6:**
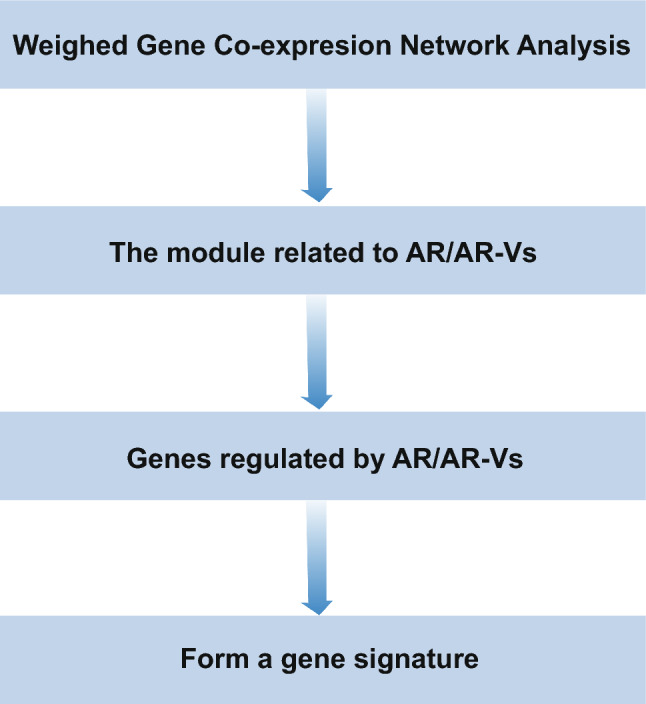
The flow chart of strategy III

Due to the importance of AR (androgen receptor) and AR-Vs (androgen receptor variants) in PC, the establishment of gene signatures related to AR or AR-Vs was considered as Strategy III. The effect of AR and AR-Vs on PC cells [36–39] was shown in Fig. 5. Androgen receptors play an important role in the development of both normal and cancerous prostate tissue by regulating proliferation-related genes expression [40] (Fig. 5). Therefore, researchers have tried to identify genes related to survival events and regulated by AR as PC biomarkers and therapy targets. The major steps of Strategy III workflow were shown in Fig. 6. Chen X et al. (2019) identified 29 gene modules using Weighted Gene Co-expression Network Analysis (WGCNA) method, and the biological function of the module significantly regulated by AR is “generation of precursor metabolites and energy” [41]. Eleven genes in this module are involved in this biological function, among which FECH and CROT are regulated by androgen and CROT has androgen receptor binding sites. Finally, a 2-gene signature was established to predict recurrence (RFS), and notably blocking this AR-related biological process will contribute to preventing PC from malignant progression [41].

Androgen Deprivation Therapy (ADT) is a preferred treatment for patients with PC metastasis [42]. However, some patients developed androgen resistance after treatment [43]. Mechanisms of androgen resistance may include AR variants splice, AR overexpression and alterations in AR coregulators [44, 45]. Therefore, Magani F et al. (2018) focused on AR splice variants, and discovered gene modules associated with different phenotypes of PC using WGCNA method [38]. As a result, most genes in one module were regulated by AR-V7 (an AR splice variant), and the main biological functions of this module were “cell proliferation” and “chromosome segregation”. Moreover, a 7-gene (KIF20A, KIF23, TOP2A, CCNB1, CCNB2, BUB1, BUB1B) signature with predictive value was constructed by further analysis of this module [38].

Through the construction of AR or AR-Vs related gene signature, not only provide effective prognosis prediction models for PC patients, but also help to elucidate the molecular mechanism underlying the occurrence and progression of PC, and will potentially facilitate the development of prognostic biomarkers and molecular targeted therapy strategies for PC. More gene signature related information of Strategy III was presented in Table 3.

### Other strategies

Although we have concluded three common strategies, other approaches for establishing gene signature were also mentioned. Li X et al. (2020) performed univariate Cox regression analysis (FDR < 20%) and screened out 80 prognosis-related genes [46]. 74 pairs of genes were identified as a gene signature according to C index. Patients with at least 37 pairs of high-risk genes were considered as high-risk group, and those with low-risk genes were considered as low-risk group [46]. Shi R et al. (2019) divided genes into co-expression modules, selected the modules significantly correlated with BFS by Cox regression analysis, and then performed LASSO regression analysis for screening genes to obtain a gene signature [47]. Cho H et al. (2021) analyzed the circulating tumor cells (CTC) and formed a gene signature derived from the representative genes in CTC [48]. More information of gene signatures derived from other strategies were exhibited in Table 4.

### Identification of robust genes in 71 different gene signatures

**Fig. 7:**
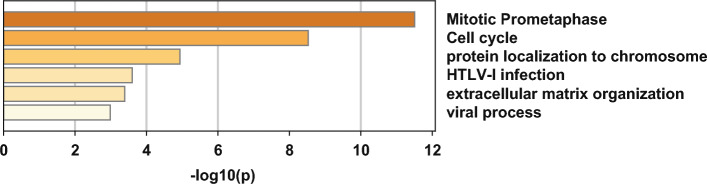
Pathway and process enrichment analysis of 14 robust genes

In 71 different gene signatures, certain genes exhibited the high frequency of application, which were considered as robust genes with excellent predictive abilities. After screening, we found that 71 gene signatures included 1278 genes, among which 381 genes were employed more than or equal to twice; 41 genes more than or equal to three times; 14 genes more than or equal to four times (regarded as robust genes) (Table 5). Furthermore, pathway and process enrichment analysis of 14 robust genes (ASPN, BIRC5, BUB1, BUB1B, CCNB1, CDK1, CENPF, DPP4, EZH2, MYH11, POSTN, PTTG1, SMC4, ZFP36) showed that these genes were mainly enriched in: Mitotic prometaphase; Cell cycle; protein localization to chromosome (Fig. 7).

**Table 5:** The summary of 14 robust genes

Gene symbol	Full name	Biological function	References	Frequency
ASPN	Asporin	Negatively regulates chondrogenesis in the articular cartilage	Planche Anne et al. [55] Sinnott Jennifer et al. [56] Jhun Min et al. [67] Li Xiang et al. [46]	4
BIRC5	Baculoviral IAP repeat containing 5	Promote cell proliferation and prevent apoptosis	Gu Yan et al. [6] Hu Daixing et al. [33] Ragnum H et al. [75] Mazzu Ying et al. [91] CCP[29, 76, 77]	5
BUB1	BUB1 mitotic checkpoint serine/threonine kinase	Correct chromosome alignment	Chipidza Fallon et al. [6] Jhun Min et al. [67] Magani Fiorella et al. [38] Glinsky Gennadi et al. [90]	4
BUB1B	BUB1 Mitotic checkpoint serine/threonine kinase B	Inhibit the activity of the anaphase-promoting complex/cyclosome (APC/C) by blocking the binding of CDC20 to APC/C	Rajan Prabhakar et al. [52] Georgescu Constantin et al. [65] Magani Fiorella et al. [38] CCP[29, 76, 77]	4
CCNB1	Cyclin B1	Control the cell cycle at the G2/M (mitosis) transition	Rajan Prabhakar et al. [52] Magani Fiorella et al. [38] Glinsky Gennadi et al. [90] Mazzu Ying et al. [91]	4
CDK1	Cyclin dependent kinase 1	Plays a key role in the control of the eukaryotic cell cycle	Rajan Prabhakar et al. [52] Georgescu Constantin et al. [65] Zhang Yao et al. [78] Zhang Yao et al. [78]	4
CENPF	Centromere protein F	Required for kinetochore function and chromosome segregation in mitosis	Zhao ShuangG et al. [15] Chipidza Fallon et al. [6] Li Ping et al. [66] Jhun Min et al. [67] CCP[29, 76, 77] Mazzu Ying et al. [91]	6
DPP4	Dipeptidyl peptidase 4	Acts as a positive regulator of T-cell coactivation	Wu Chin-lee et al. [68] Sinnott Jennifer et al. [56] Zhao yue et al. [62] Walker Steven et al. [92]	4
EZH2	Enhancer Of Zeste 2 polycomb repressive complex 2 subunit	Methylates 'Lys-9' (H3K9me) and 'Lys-27' (H3K27me) of histone H3	Xiao Kefeng et al. [19] Xiao Kefeng et al. [19] Chipidza Fallon et al. [6] Yuan Penghui et al. [24] Karnes R et al. [88]	5
MYH11	Myosin heavy chain 11	Muscle contraction	Sinnott Jennifer et al. [56] Mu Haiqi et al. [50] Ong Chee et al. [26] Zhang Shengping et al. [84]	4
POSTN	Periostin	Induces cell attachment and spreading and plays a role in cell adhesion	Zhao ShuangG et al. [15] Planche Anne et al. [55] Pang Xiaocong et al. [27] Li Xiang et al. [46]	4
PTTG1	PTTG1 regulator Of sister chromatid separation, securin	Plays a central role in chromosome stability, in the p53/TP53 pathway, and DNA repair	Chipidza Fallon et al. [6] CCP[29, 76, 77] Mazzu Ying et al. [91] Walker Steven et al. [92]	4
SMC4	Structural maintenance Of chromosomes 4	Central component of the condensin complex	Zhao ShuangG et al. [15] Chipidza Fallon et al. [6] Jhun Min et al. [67] Zhang Yao et al.[78] Zhang Yao et al.[78]	5
ZFP36	ZFP36 ring finger protein	Destabilize mRNA transcripts	Sinnott Jennifer et al. [56] Yang Lingjian et al. [70] Li Xiang et al. [46] Walker Steven et al. [92]	4

*CCP* gene originate from 31 CCP (cell cycle progression) genes

## Discussion

In the present study, we conducted a systematic review and integrated analysis of previously reported prognostic gene signatures of PC. Gene signature construction strategies and robust genes significantly associated with prognosis were summarized. First, the reported gene signatures were collected and sorted; second, three strategies for gene signature establishment were summarized and the information of prognostic abilities of gene signatures was listed and exhibited as each strategy; third, the robust genes were identified from the reported gene signatures with risk prediction abilities.

In the process of constructing gene signature of PC, biochemical recurrence (BCR) and castration-resistant prostate cancer (CRPC) have been introduced to be evaluated. The majority of patients with early-stage PC were treated with prostatectomy. However, about one-third of patients will develop BCR after surgical treatment, leading to the poor prognosis of them [6, 7]. So it is required for gene signatures acting as predictors of BCR to discriminate patients at high risk. In addition to BCR, CRPC is also a challenge in PC treatment. Urbanucci A et al. (2017) demonstrated that overexpressed androgen receptors lead to chromatin relaxation, which are thought to be involved in CRPC by regulating bromodomain-containing proteins (BRDs) [39]. Furthermore, Zhang Q et al. (2020) [30], Peng Z et al. (2016) [49], Chen X et al. (2012) [14], and Yuan P et al. (2020) [24] showed that the predictive ability of a gene signature in combination with clinical characteristics (e.g. Gleason score) was stronger than that of a gene signature alone. In addition, Zhao SG et al. (2016) [15], Mu HQ et al. (2020) [50], and Pang X et al. (2019) [27] mentioned that genes in their signatures have a certain relationship with drug therapy, indicating that these gene signatures for PC might help identify potential therapeutic targets. With the development of bioinformatics, more and more researchers have applied GO or pathway enrichment analysis and protein network into the study of gene signature to better meet the research purpose [16, 24, 51].

## Data Availability

The data supporting this systematic review were derived from previously reported studies, which were collected from PubMed database and have been cited. The processed data are available from corresponding author upon request.

## Declarations

### Code availability

Source code is available upon request from the corresponding author.
